# Subnormothermic Machine Perfusion of Steatotic Livers Results in Increased Energy Charge at the Cost of Anti-Oxidant Capacity Compared to Normothermic Perfusion

**DOI:** 10.3390/metabo9110246

**Published:** 2019-10-24

**Authors:** Negin Karimian, Siavash Raigani, Viola Huang, Sonal Nagpal, Ehab O. A. Hafiz, Irene Beijert, Paria Mahboub, Robert J. Porte, Korkut Uygun, Martin Yarmush, Heidi Yeh

**Affiliations:** 1 Center for Engineering in Medicine, Massachusetts General Hospital, Harvard Medical School, Boston, MA 02115, USA; n.karimian284@gmail.com (N.K.); sraigani@partners.org (S.R.); vhuang450@gmail.com (V.H.); nsonal400@gmail.com (S.N.); paria.mahboub@gmail.com (P.M.); KUYGUN@mgh.harvard.edu (K.U.); MYARMUSH@mgh.harvard.edu (M.Y.); 2 Division of Transplant Surgery, Massachusetts General Hospital, Harvard Medical School, Boston, MA 02115, USA; 3 Shriners Hospital for Children, Boston, MA 02114, USA; 4 Electron Microscopy Research Department, Theodor Bilharz Research Institute, 12411 Giza, Egypt; dr_ehabosama@yahoo.com; 5 Division of Hepatobiliary Surgery and Liver Transplantation, University Medical Center Groningen, 9700 Groningen, The Netherlands; ibie65552@gmail.com (I.B.); r.j.porte@umcg.nl (R.J.P.); 6 Department of Biomedical Engineering, Rutgers University, Piscataway, NJ 08854, USA

**Keywords:** steatosis, subnormothermic, normothermic, machine perfusion, anti-oxidant, metabolomics, liver transplantation

## Abstract

There continues to be significant debate regarding the most effective mode of ex situ machine perfusion of livers for transplantation. Subnormothermic (SNMP) and normothermic machine perfusion (NMP) are two methods with different benefits. We examined the metabolomic profiles of discarded steatotic human livers during three hours of subnormothermic or normothermic machine perfusion. Steatotic livers regenerate higher stores of ATP during SNMP than NMP. However, there is a significant depletion of available glutathione during SNMP, likely due to an inability to overcome the high energy threshold needed to synthesize glutathione. This highlights the increased oxidative stress apparent in steatotic livers. Rescue of discarded steatotic livers with machine perfusion may require the optimization of redox status through repletion or supplementation of reducing agents.

## 1. Introduction

Liver transplantation remains the only definitive cure for end-stage liver disease. Despite significant success and innovation in the field, there continues to be a severe shortage of organs that is unable to meet the need of transplant candidates. Machine perfusion technology has the potential to alleviate a significant portion of the shortage, with initial clinical trial results demonstrating lower organ discard rates in livers preserved by normothermic machine perfusion (NMP) compared to conventional static cold storage (SCS) [1].

Machine perfusion has been incorporated into clinical practice by transplant centers around the world. However, there is a significant range of perfusion practices among different centers. Groups have reported their experience with NMP [2,3,4], subnormothermic machine perfusion (SNMP) [5,6,7], and hypothermic oxygenated perfusion (HOPE) [8,9], each with its own unique benefits and deficits. NMP allows surgeons to evaluate the viability of grafts in real-time under conditions that most closely simulate transplantation [2]. In addition, NMP can be leveraged as a platform for therapeutic interventions that aim to remodel or rehabilitate marginal grafts [10]. SNMP and HOPE repair mitochondrial injury and maximize adenosine triphosphate (ATP) stores prior to transplantation, though viability assessment is not currently well-defined below normothermic temperatures [6,11]. Additionally, there is the added benefit of not needing to use an oxygen carrier, such as packed red blood cells, at lower perfusion temperatures. Recently, perfusion techniques have been combined in an attempt to maximize the benefits of each method [12,13]. Currently, there is no consensus on the best approach for machine perfusion of livers. This is further complicated by the heterogeneity of donor livers, which include donation after cardiac death (DCD), steatotic grafts, and other extended-criteria (or marginal) donor characteristics, each of which may benefit from a different application of machine perfusion.

Further elucidation of these subsets of donor grafts and their physiology during machine perfusion is required to allow informed decision making by transplant surgeons. We compared the metabolomic profiles of steatotic livers perfused at subnormothermic versus normothermic temperatures to evaluate the advantages and disadvantages of each.

## 2. Results

### 2.1. Functional Recovery during Perfusion

**Figure 1:**
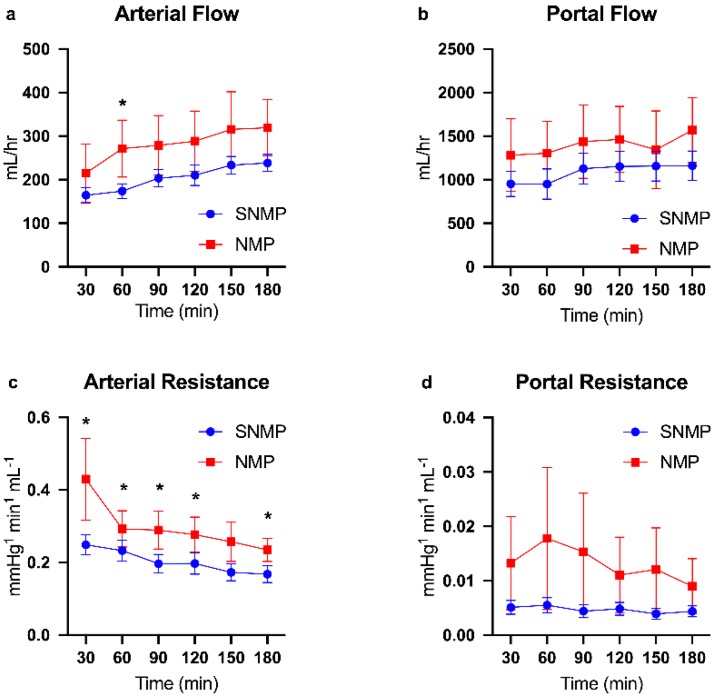
Perfusion parameters during machine perfusion of steatotic livers. Steatotic livers had higher qualitative hepatic artery (**a**) and portal vein (**b**) flows during NMP, though this was not statistically significant. Arterial resistance (**c**) was significantly higher throughout the majority of three hours perfusion during NMP compared to SNMP. Portal vein resistance (**d**) was also higher during NMP but not statistically significant due to large variance. Random intercept mixed model analysis for longitudinal data was used. Data shown as mean ± SEM, * indicates *p* < 0.05. NMP, normothermic machine perfusion; SNMP, subnormothermic machine perfusion.

**Figure 2:**
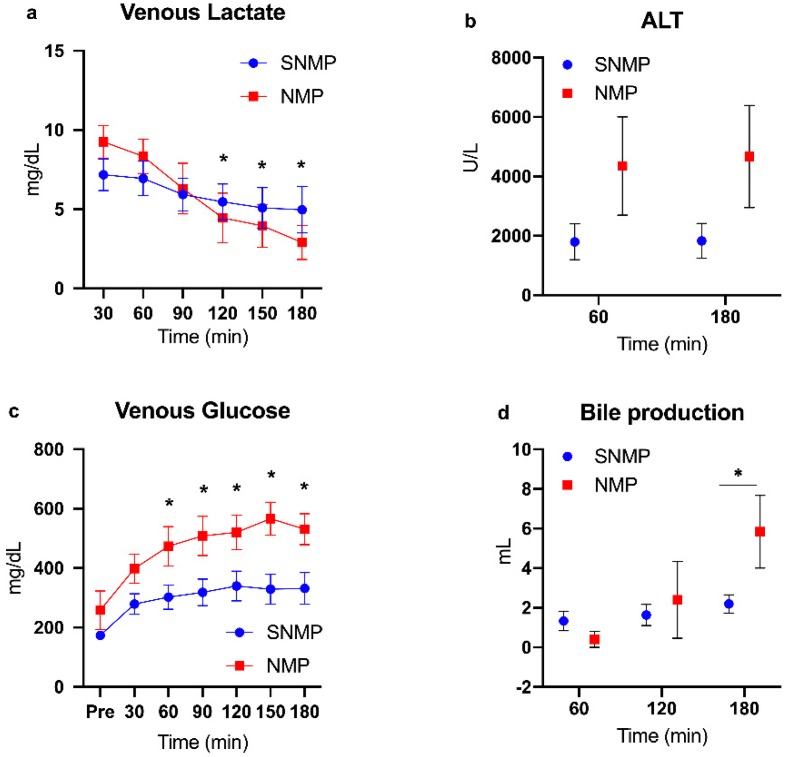
Functional outcome measures during machine perfusion of steatotic livers. NMP of steatotic livers resulted in evidence of higher metabolic activity as expected compared to SNMP. Venous lactate content (**a**) decreased during three hours of perfusion in both groups but at a fast rate during NMP. ALT (**b**) is qualitatively higher in steatotic livers during NMP compared to SNMP but did not reach significance. Glucose content in the perfusate (**c**) was significantly higher during NMP. Steatotic livers produced comparable volumes of bile during the first 2 h of perfusion, but production was significantly higher by the third hour compared to SNMP (**d**) Data shown as mean ± SEM, * indicates *p* < 0.05. NMP, normothermic machine perfusion; SNMP, subnormothermic machine perfusion; ALT, alanine aminotransferase.

Donor demographics are presented in Table 1. Given the temperature and perfusate differences between the experimental arms, expected differences in perfusion dynamics were observed. NMP livers demonstrated higher flow rates and resistance in both arterial and portal vein circuits (Figure 1a–d). NMP livers also cleared lactate at a faster rate compared to SNMP livers, which became significant after 120 min of perfusion in the mixed model. Similarly, NMP livers produced more bile with increasing perfusion time, demonstrating a significant difference at 180 min. Glucose levels in the perfusate were significantly higher in the NMP group after 60 min of perfusion compared to the SNMP group. Finally, ALT levels were higher in NMP livers at 60 (4350 ± 1656 U/L vs. 1800 ± 602, *p =* 0.35) and 180 min (4672 ± 1721 vs. 1831 ± 587, *p =* 0.25) of perfusion, though they did not reach significance (Figure 2a–d).

### 2.2. Histologic Assessment

**Figure 3:**
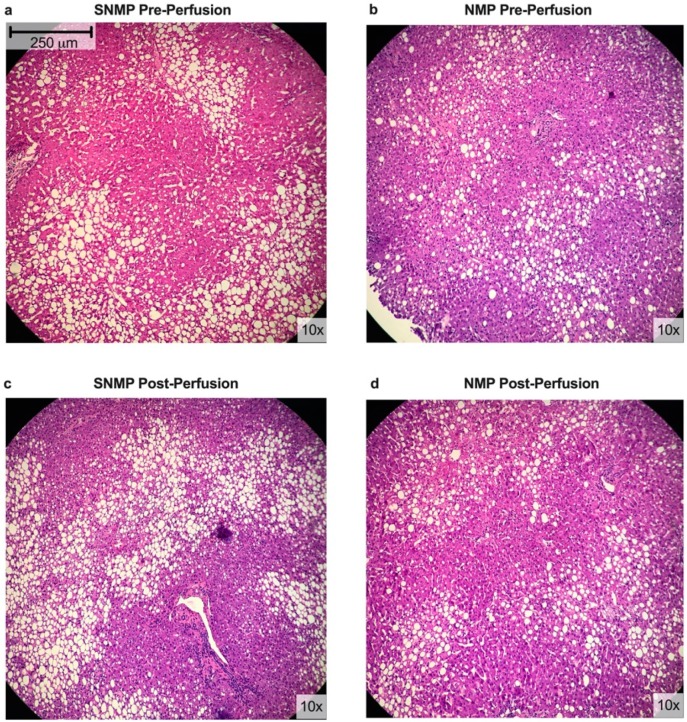
Histologic differences of steatotic livers during NMP and SNMP. Representative H&E-stained liver sections from steatotic livers undergoing NMP and SNMP shown pre-perfusion and post-perfusion. No significant change in macrosteatosis was observed with either perfusion modality. (**a**) pre-perfusion biopsy of liver in SNMP group, (**b**) pre-perfusion biopsy of liver in NMP group, (**c**) biopsy after 3 hours of SNMP, (**d**) biopsy after 3 hours of NMP.

Figure 3 shows representative histology for liver biopsies from both experimental groups. Comparison of pre- and post-perfusion histology demonstrates well-preserved architecture with minimal swelling and patent sinusoids in SNMP livers. NMP livers had similar findings, with preserved liver lobules, portal tract structures, and mild congestion of sinusoids. Table S2 lists the macrosteatotic content of each liver pre- and post-perfusion.

### 2.3. Targeted Energy Cofactor Analysis 

**Figure 4:**
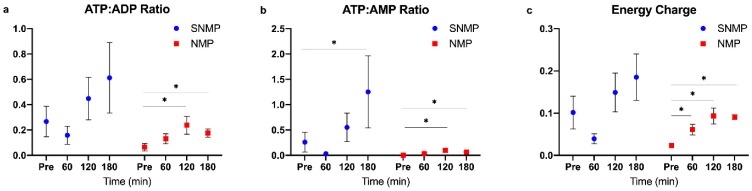
Energy cofactor changes during NMP and SNMP of steatotic livers. (**a**) ATP:ADP, (**b**) ATP:AMP, and (**c**) energy charge ratios at each hour of perfusion. Given the lower metabolic activity at subnormothermic temperatures, steatotic livers are able to regenerate ATP and energy charge to higher levels with oxygenated perfusion compared to NMP. Energy charge was calculated as (ATP + ADP*0.5)/(ATP + ADP + AMP). Within group comparisons at 60, 120, and 180 min are made to a pre-perfusion measurement (time = 0 min). * indicates *p* < 0.05 for random intercept mixed model comparison of longitudinal data to pre-perfusion levels. Data shown are mean ± SEM. NMP, normothermic machine perfusion; SNMP, subnormothermic machine perfusion.

Livers undergoing SNMP and NMP demonstrated significant increases in ATP levels following three hours of perfusion. Despite not reaching significance in ATP:ADP and energy charge ratios, SNMP livers demonstrated a qualitatively larger increase in ATP stores compared to NMP livers. The ATP:AMP ratio was significantly higher at 180 min compared to pre-perfusion levels in the SNMP group, demonstrating a ratio >1. Interestingly, SNMP livers experienced a drop in ATP stores after 60 min of perfusion but were able to regenerate this supply with ongoing perfusion. The more metabolically active NMP livers demonstrated smaller but significant increases in ATP:ADP, ATP:AMP, and energy charge ratios after 120 and 180 min of perfusion compared to pre-perfusion levels (Figure 4). 

### 2.4. Untargeted Metabolomic Analysis

#### 2.4.1. Data Reduction Methods

**Figure 5:**
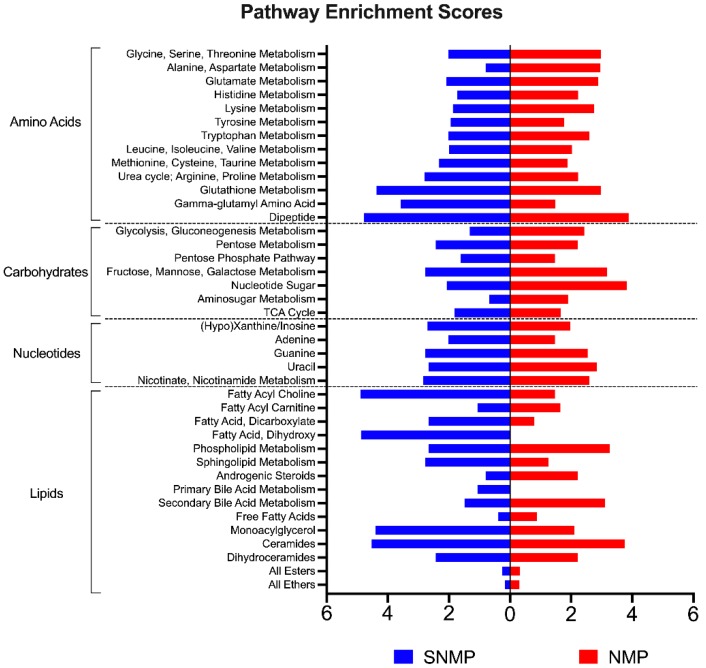
Pathway enrichment analysis for metabolomic profiles of steatotic livers during NMP and SNMP. Pathway enrichment scores of metabolite subgroups at 180 min of perfusion compared to pre-perfusion concentrations between SNMP and NMP groups. NMP, normothermic machine perfusion; SNMP, subnormothermic machine perfusion.

Figure 5 illustrates pathway enrichment scores for comparisons of tissue metabolite ratios for SNMP and NMP livers (*n* = 3 each) at 180 min compared to pre-perfusion concentrations. Subsequently, principle component analysis (PCA) demonstrated similar composition of metabolites in pre-perfusion biopsies of steatotic livers, with divergence after initiation of SNMP or NMP. Interestingly, PCA of lipids clustered by mode of perfusion (Figure S1). 

#### 2.4.2. Anti-Oxidative Capacity

**Figure 6:**
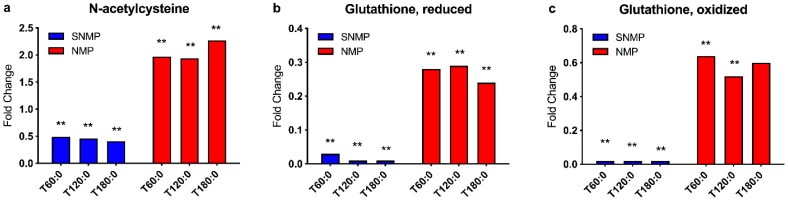
Redox status during NMP and SNMP of steatotic Livers. (**a**) N-acetylcysteine, (**b**) reduced glutathione, and (**c**) oxidized glutathione ratios demonstrate depleted redox capacity within hepatocytes during SNMP compared to NMP. ** indicated *p* < 0.05. NMP, normothermic machine perfusion; SNMP, subnormothermic machine perfusion; x-axis represents fold change at 60, 120, and 180 min compared to pre-perfusion concentrations.

A significantly different anti-oxidative capacity was seen in the two groups. SNMP livers demonstrated a reduction in tissue N-acetylcysteine (N-Ac) levels (fold change range 0.41–0.49, *p* < 0.05 for all), the main cysteine donor involved in glutathione synthesis. NMP livers, however, demonstrated a gain in N-Ac stores during three hours of perfusion (fold change range 1.94–2.27, *p* < 0.05 for all). Notably, there was marked depletion of tissue reduced glutathione (GSH) levels in SNMP livers (fold change range 0.01–0.03, *p* < 0.05 for all), suggesting the immediate utilization of available glutathione after initiation of SNMP. Oxidized glutathione (GSSG) levels decreased to a ratio of 0.02 at 60 min of perfusion from pre-perfusion levels (*p* < 0.05), indicating degradation and utilization of glutathione components for other cellular processes. NMP livers were able to sustain their anti-oxidative capacity comparatively; GSH levels decreased after the initiation of perfusion (0.24–0.29, *p* < 0.05 for all) but to a lesser degree than SNMP livers (Figure 6a–c). 

#### 2.4.3. Glutathione Cycle

**Figure 7:**
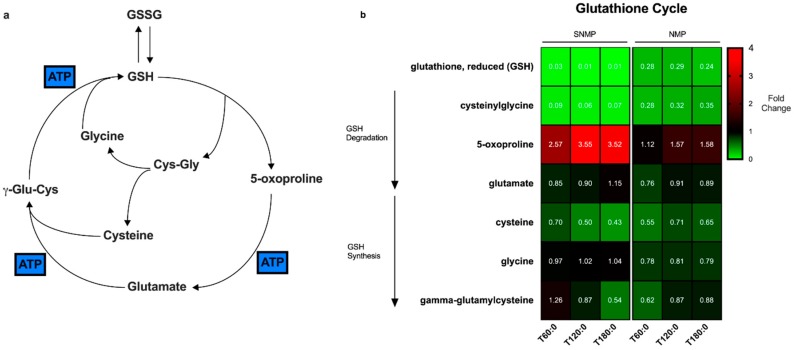
Glutathione degradation varies in steatotic livers during SNMP compared to NMP. (**a**) The glutathione cycle involves the degradation of glutathione into its base amino acids. Synthesis of glutathione involves three molecules of ATP. (**b**) Heatmap of the metabolites in the glutathione cycle compared between SNMP and NMP of steatotic livers. There is a significant depletion of the precursor metabolites in the synthesis arm of the cycle during SNMP compared to NMP, as well as a larger accumulation of 5-oxoproline. Reduced glutathione content in hepatocytes is decreased during NMP but depletion is near-total during SNMP. GSH, reduced glutathione; GSSH, oxidized glutathione; Cys-Gly, cysteinylglycine; γ-Glu-Cys, γ-glutamylcysteine; NMP, normothermic machine perfusion; SNMP, subnormothermic machine perfusion; x-axis represents fold change at 60, 120, and 180 min compared to pre-perfusion concentrations.

The glutathione cycle is the degradation and biosynthetic pathway by which glutathione is metabolized in cells (Figure 7a). GSH is regenerated at the expense of three molecules of ATP [14]. Figure 7b represents a heatmap of the metabolites involved in the glutathione cycle. SNMP livers demonstrate near-total depletion of GSH compared to NMP livers. The precursor metabolites for glutathione biosynthesis (cysteine, γ-glutamylcysteine) are similarly depleted over time to a larger degree in SNMP vs. NMP livers. During the same interval, there is a significant accumulation of the GSH breakdown product, 5-oxoproline, and to a lesser extent, glutamate, as the degradation of each metabolite requires one molecule of ATP.

#### 2.4.4. Carbohydrate Metabolism

NMP livers demonstrated qualitatively higher ratios of glycogen components with perfusion compared to SNMP livers. One component, maltose, reached significance with fold change ratios 2.27 and 2.89 (*p* < 0.05 for both) at 120 and 180 min of perfusion, respectively. Metabolite concentrations involved in the tricyclic acid cycle generally showed increased fold change ratios in SNMP livers and decreased ratios in NMP livers, consistent with the temperature-dependent rate of metabolic activity (Figure S2).

#### 2.4.5. Lipid Metabolism

Several subclasses of lipid metabolites demonstrated notable enrichment scores during SNMP. Interestingly, fatty acyl cholines, hydroxyeicosatrienoic acids (cytochrome P450 eicosanoids), and dicarboxylic fatty acids demonstrated significantly increased tissue concentrations with perfusion compared to NMP livers. Ceramides and dihydroceramides were significantly increased in both groups, while sphingolipids and monoacylglycerols were significantly decreased in SNMP livers (Supplemental Datasheet).

## 3. Discussion

The debate regarding the best modality of machine perfusion continues. This study provides a metabolomic profile of discarded steatotic human livers during three hours of subnormothermic versus normothermic machine perfusion to help inform future perfusion studies. 

Our most significant finding is that steatotic livers undergoing SNMP are able to increase ATP and energy charge ratios; however, this appears to hinder the anti-oxidative capacity that would be required to overcome the oxidative injury encountered during surgical reperfusion at the time of implantation [15]. Given that metabolic activity and consumption of ATP is decreased at lower organ temperatures [16], the accumulation of ATP during SNMP is not surprising. Conversely, while mitochondrial activity is higher at normothermic temperatures, so is ATP consumption through energy-driven processes. Unfortunately, the regeneration of ATP during SNMP is associated with glutathione depletion, and while they may not be directly related, do still represent a significant trade-off between the benefits of SNMP compared to NMP. Glutathione degradation has until now been an unrecognized disadvantage of SNMP of steatotic human livers. The inability of hepatocytes to synthesize glutathione is likely responsible for the massive accumulation of its breakdown product and precursor, 5-oxoproline. This may lead to further inherent cytotoxicity of the graft as the accumulation of 5-oxoproline has been associated with hemolytic anemia, metabolic acidosis, and central nervous system damage in inherited glutathione synthetase deficiency [17]. Residual metabolic activity at subnormothermic temperature allows some progression of 5-oxoproline through the first ATP-dependent step to glutamate, which accumulates to a much lesser extent than 5-oxoproline. Further, ATP-dependent advancement to glutathione regeneration appears to be significantly impaired, with marked depletion of subsequent intermediates, such that the immediate precursor of glutathione, γ-glutamylcysteine (and breakdown product of glutamate) dramatically decreases during SNMP. This is in direct contrast to NMP, where γ-glutamylcysteine concentrations increase during perfusion and glutathione stores are able to be maintained. This is a clinically relevant finding given prior studies demonstrating an acute depletion of reduced glutathione stores following graft reperfusion in clinical liver transplantation studies [18,19]. Though no clinical reports of transplantation of steatotic human livers after SNMP exist, these grafts may suffer severe ischemic-reperfusion injury (IRI) at reperfusion and could potentially experience higher rates of early allograft dysfunction (EAD) or post-reperfusion syndrome (PRS) as a result of glutathione depletion. Interestingly, even oxidized glutathione stores are depleted during SNMP, which may indicate the breakdown of glutathione for its essential amino acids (cysteine) to be used in other cellular processes. 

One potential way to overcome glutathione depletion while capitalizing on ATP production is to combine perfusion modalities. Several groups have reported their experience with combined hypothermic and normothermic machine perfusion of livers with results indicating improved liver function and viability compared to NMP or SCS alone [13,20]. This technique and a similar one, known as controlled oxygenated rewarming, have shown promise in animal and human trials [21,22]. An initial period of hypothermic perfusion, with or without an acellular oxygen carrier, allows the regeneration of ATP stores, followed by a period of normothermic perfusion for viability assessment. Similar strategies with SNMP preceding NMP could provide the benefits of both modalities. Alternatively or in addition, supplementation of the perfusate with anti-oxidants, such as glutathione [23], N-acetylcysteine [24], or Vitamin E [25], could buffer against the depletion of glutathione seen during SNMP.

With respect to lipid metabolism, it is notable that steatotic livers appear to synthesize ceramides regardless of perfusion modality. Ceramides can be generated through the hydrolysis of sphingomyelin by the enzyme acid sphingomyelinase (ASMase) or through de novo synthesis in the endoplasmic reticulum. ASMase appears to be linked to key mechanisms involved in the regulation of steatosis, fibrosis, and lipotoxicity [26]. The inhibition of de novo ceramide synthesis has been demonstrated to decrease hepatic steatosis [27] and improve insulin resistance [28] in animal models, suggesting that either temperature may be efficacious in strategies targeting lipid modulation.

In contrast, SNMP livers demonstrate significantly larger fold increases in cytochrome P450 (CYP450) eicosanoid products, 14,15-dihydroxyeicosatrienoic acid (14,15-DHET or 14,15-DiHETrE) and 8,9-dihydroxyeicosatrienoic acid (8,9-DHET). DHETs are the breakdown product of epoxyeicosatrienoic acids (EETs), which have been shown to possess significant vasodilatory and anti-inflammatory properties and play an important role in liver physiology. One limitation of our metabolomic analysis is that it does not include the concentrations of EETs or its counterpart 20-hydroxyeicosatraenoic acid (20-HETE), which has vasoconstrictive and pro-inflammatory properties [29], but if the breakdown product, DHETs, are found in higher concentrations during SNMP, this could indicate that anti-inflammatory or vasoactive supplements are more important during SNMP than NMP. Pharmacologic manipulation of CYP450 eicosanoid metabolism is an active area of drug investigation in cardiovascular disease [30,31] and has a potentially important and novel application in machine perfusion of steatotic liver grafts by improving the hindered microcirculation and decreasing pro-inflammatory signaling [32] associated with ischemia-reperfusion injury. 

Several limitations are worth discussion. First, there is inherent heterogeneity in discarded organ research. Livers with similar profiles were submitted for metabolomic analysis in an attempt to control for these various factors, though variability was unavoidable. Notably, the two groups differed in macrosteatosis (more severe macrosteatosis in SNMP group compared to NMP), which could impact the functional and metabolomic changes observed. In addition, baseline comparison of lipidomic profiles prior to initiation of perfusion demonstrated pathway enrichment in phospholipid and secondary bile acid metabolism. Though the majority of the lipid subclasses demonstrated no enrichment, these pre-perfusion differences could certainly impact the observed changes during perfusion (Figure S3). This study also used a synthetic hemoglobin-based oxygen carrier rather than conventional packed red blood cells, given evidence of similar efficacy [3]. While the use of a blood-based oxygen carrier would have impacted the metabolic profiles of the perfused livers, the use of HBOC-201 may provide a more consistent picture of metabolic activity, has the benefits of avoiding consequences of pooled blood donations (such as RBC hemolysis and immune-mediated phenomena) [33], and is capable of being perfused at hypothermic, subnormothermic, and normothermic temperatures [13]. While the use of HBOC-201 is likely to minimize experimental variability, some metabolic differences should be expected if a blood-based perfused is used, therefore future comparisons with literature data should be done with caution. The use of fresh frozen plasma and albumin in the NMP perfusate does represent a significant procedural difference compared to the SNMP perfusate, which resulted in the lower vascular resistance seen in the SNMP livers. Future studies should ideally avoid the use of FFP in favor of using albumin for the benefit of oncotic pressure. Finally, we performed a three-hour perfusion, as this is generally the clinical timeframe in which transplant surgeons determine viability of a machine perfused graft [2], and prior studies have shown that livers achieve a physiologic steady-state by three hours [6,34,35]. This allowed the metabolic differences between SNMP and NMP of steatotic grafts to be well-defined in order to achieve our goal of optimizing these grafts for transplantation within a relevant time period, however analysis of metabolic changes over a longer perfusion period in future studies will strengthen these results. 

The use of metabolomics has numerous clinically relevant applications for transplantation. In this study, metabolomic profiling using mass spectrometry was used to examine differences between discarded livers undergoing SNMP versus NMP in order to assist in designing optimized perfusion conditions for steatotic grafts. Faitot et al. reported the use of real-time high-resolution nuclear magnetic resonance (NMR) metabolomic analysis in patients undergoing liver transplantation and demonstrated the ability to accurately predict EAD using lactate and phosphocholine (L + PC) levels [36]. Notably, the authors describe that metabolic mismatch between the recipient native liver and donor graft L + PC levels resulted in 100% incidence of EAD, indicating that donor-recipient matching of metabolic profiles has a significant role in graft function after implantation. Incorporating machine perfusion and real-time NMR into the transplant workflow can further enhance the surgeon’s ability to match metabolic profiles of donor and recipient, as grafts with mismatched profiles can potentially be further optimized with targeted perfusion techniques in a realistic timeframe. This may be a novel application of both technologies with real potential to bring bench research into the operating room with the goal of improving patient outcomes.

## 4. Materials and Methods

### 4.1. Donor Livers

Fourteen human donor livers with moderate (30–59%) or severe (>60%) macrosteatosis, declined for transplantation by all transplant centers in our donation service area, with consent for research, between August 2015 and April 2018 were included in this study. All donor livers were received through New England Donor Services (NEDS); no organs were procured from prisoners. Donors or their surrogates (including parents or legal guardians) provided informed consent for use of donor organs in research. The Massachusetts General Hospital Institutional Review Board (IRB) and the NEDS approved this study (No. 2011P001496), and all studies were carried out in accordance with IRB and NEDS approved guidelines. 

### 4.2. Procurement and Preparation of Liver Grafts

All donor livers were procured based on the standard technique of in situ cold flush using University of Wisconsin (UW) preservation solution. Procurement techniques for donation after brain death (DBD) or donation after circulatory death are previously described [6]. Warm ischemic time (WIT) is defined as time from circulatory arrest to in situ cold flushing for DCD livers. Cold ischemic time (CIT) is defined from in situ cold flushing to start of machine perfusion. 

Upon arrival to our perfusion lab, back table preparation of the livers was performed as described previously [37]. 

### 4.3. Machine Perfusion

Liver grafts were perfused for three hours using a pressure and temperature-controlled perfusion device, Liver Assist (Organ Assist, Groningen, Netherlands). During SNMP, livers were perfused with a portal venous pressure (PVP) of 3–7 mmHg and mean hepatic arterial pressure (HAP) of 30–60 mmHg. The temperature of the perfusate was maintained between 20–22 °C. The perfusate consisted of Williams’ medium E supplemented with hydrocortisone, insulin, and penicillin/streptomycin [6]. During NMP, albumin, fresh frozen plasma and a hemoglobin-based oxygen carrier, HBOC-201 (HbO_2_ Therapeutics LLC, Souderton, PA, USA) were added to the base perfusate. Detailed composition is provided in the Supplementary Methods (Table S2). NMP grafts were perfused at a PVP 6–8 mm Hg and HAP 60–70 mm Hg. Perfusate samples were collected from the arterial inflow and venous outflow at 30-min intervals. Blood gas and chemistry analysis was performed using i-STAT Blood Analyzer (Abbott Point of Care Inc., Princeton, NJ, USA). Donor livers were perfused for three hours, a duration chosen based on prior studies demonstrating adequate ATP regeneration within that time period [6,38].

### 4.4. Evaluation of Hepatic Injury and Function

Perfusion dynamics were recorded every 30 min. Vascular resistance was defined as perfusion pressure divided by flow rate (mmHg^1^ min^1^ mL^−1^). Alanine aminotransferase (ALT) was determined using Piccolo Xpress Chemistry Analyzer (Abbott Point of Care Inc., Princeton, NJ, USA). The volume of bile produced was recorded at 1-h intervals. Two wedge liver biopsies were collected immediately prior to perfusion and then hourly during perfusion. One tissue sample was snap-frozen in liquid nitrogen for future analysis and the other preserved in formalin for histology. 

### 4.5. Histological Assessment

After formalin fixation, tissue samples were paraffin-embedded, and stained with hematoxylin-eosin (H&E). Tissue slides were evaluated for macrosteatosis content and preservation injury by a blinded expert pathologist (EOAH). 

### 4.6. Targeted Metabolomics Analysis—Energy Cofactors

Frozen liver biopsies from each time point (~25 mg) were pulverized in liquid nitrogen and analyzed for metabolic cofactors using a targeted multiple reaction monitoring analysis on a Sciex TripleTOF 6600 Quadruple Time-Of-Flight system, performed at the principle research institution. Metabolites were extracted using the protocol provided by Yuan et al. [39]. Concentrations of hepatic adenosine tri-, di-, and mono- phosphate (ATP, ADP, AMP) were quantified. Energy charge was calculated as: [ATP + ADP*0.5] / [ATP + ADP + AMP].

### 4.7. Untargeted Metabolomic and Lipidomic Analysis 

Tissue biopsies of three livers from each group were analyzed for 1600 compounds of known identity by Metabolon, Inc. (Durham, NC, USA). Principal component analysis, pathway enrichment scores, detailed methods, and statistical approach are provided in the Supplementary Methods. 

### 4.8. Statistical Analysis

Demographic and perfusion data are presented as the mean ± standard error of the mean (SEM), unless otherwise specified, with statistical significance defined as *p* < 0.05. Wilcoxon’s rank-sum (Mann-Whitney U) test and Fischer’s exact test were used for continuous and categorical comparisons, respectively. Repeated measures data were analyzed using a random intercept mixed model with a categorical effect of time. If between-group comparisons were made, the categorical effect of group and the group by time interactions were added to the model. Statistical analysis was performed using Stata 15.1 (StataCorp, College Station, TX, USA). 

## 5. Conclusions

Subnormothermic and normothermic machine perfusion of liver grafts are techniques of organ resuscitation that produce significantly different metabolomic profiles. Perfusion protocols that combine sequential temperature variation, guided by evidence-based metabolite replacement, can maximize the potential of both methods to improve organ quality for successful transplantation. 

## Figures and Tables

**Table 1:** Donor demographics by perfusion method.

	Steatotic SNMP	Steatotic NMP
	N = 9	N = 5
Age (years)	44.9 (22–60)	48.4 (37–55)
Gender (female)	2	1
BMI (kg/m^2^)	29.4 (22.7–38)	32.6 (25.8–37.9)
Type of Donor Liver		
DCD	5	2
DBD	4	3
WIT (min)	35.3 (17–48)	27 *
CIT (min)	554 (304–808)	735 (463–935)
Weight of Liver (kg)	2.3 (1.5–3.1)	2.3 (2.0–2.8)

Mean with range in parentheses. NMP, normothermic machine perfusion; SNMP, subnormothermic machine perfusion; BMI, body mass index; DCD, donation after cardiac death; DBD, donation after brain death; WIT, warm ischemic time; CIT, cold ischemic time; * indicates one available data point. Comparisons were not statistically significant.

## Supplementary Materials

The following are available online at https://www.mdpi.com/2218-1989/9/11/246/s1: Table S1: Histologic Macrosteatosis Content of Livers Pre- and Post-Perfusion; Table S2: Perfusate Compositions; Figure S1: Principle Component Analysis of Metabolites and Lipids of Steatotic Livers during NMP and SNMP; Figure S2: Heatmap of Metabolites Involved in Tricyclic Acid Cycle and Glycogen Metabolism; Figure S3: Pathway Enrichment Analysis Comparing Pre-Perfusion Metabolomic Profiles of Steatotic Livers in SNMP versus NMP Group; Supplementary Methods: Untargeted Metabolomic and Lipidomic Data Processing and Analysis; Supplementary Datasheets: Entire metabolomic and lipidomic data set.
