# Supplementary material for: Subnormothermic Machine Perfusion of Steatotic Livers Results in Increased Energy Charge at the Cost of Anti-Oxidant Capacity Compared to Normothermic Perfusion
Source: Metabolites. 2019 Oct 24;9(11):246. doi: 10.3390/metabo9110246 (PMC6918199; doi:10.3390/metabo9110246)
Supplement: Supplementary file 1 [file metabolites-09-00246-s001.zip › Supplementary Materials.pdf]

1. Supplementary Tables

Table S1 Histologic Macrosteatosis Content of Livers Pre- and Post-Perfusion

Group	Liver #	Macrosteatosis Percentage	
		Pre-Perfusion	Post-Perfusion
	Liver #		
SNMP	1	60%	60%
	2	80%	60%
	3	60%	60%
	4	50%	60%
	5	60%	60%
	6	50%	50%
	7	60%	50%
	8	90%	90%
	9	60%	60%
NMP	1	30%	30%
	2	60%	60%
	3	30%	30%
	4	30%	30%
	5	80%	80%

NMP, normothermic machine perfusion; SNMP, subnormothermic machine perfusion

Table S2 Perfusate Compositions

SNMP Perfusate	NMP Perfusate
Williams' Medium E (2L)	Williams' Medium E (1L)
Insulin (5 U/L)	Insulin (5 U/L)
Pencillin-streptomycin (40,000 U/L)	Pencillin-streptomycin (40,000 U/L)
Hydrocortisone (10mg/L)	Hydrocortisone (10mg/L)
Sodium bicarbonate, 8.4% solution (as needed to titrate to pH 7.35-7.45)	Fresh frozen plasma (1U or ~330 mL)
	Human albumin, 25% solution (100 mL)
	Heparin (5,000 U/L)
	HBOC-201 (500 mL)
	Sodium bicarbonate, 8.4% solution (as needed to titrate to pH 7.35-7.45)

Williams' medium E (Millipore Sigma, St. Louis, MO, USA), Fresh frozen plasma (Research Blood Components, Boston, MA, USA), human albumin (CSL Behring, Kankakee, IL, USA), HBOC-201 (HbO2 Therapeutics LLC, Souderton, PA, USA), heparin (Sagent Pharmaceuticals, Schaumburg, IL, USA), hydrocortisone (Solu-Cortef, Pharmacia & Upjohn, Kalamazoo, MI,

USA), insulin (Humulin R; Eli Lilly & Co, Indianapolis, IN, USA), pencillin-streptomycin (Invitrogen, Waltham, MA, USA). SNMP, subnormothermic machine perfusion; NMP, normothermic machine perfusion.

2. Supplementary Figures

Figure S1 Principle Component Analysis of Metabolites and Lipids of Steatotic Livers during NMP and SNMP

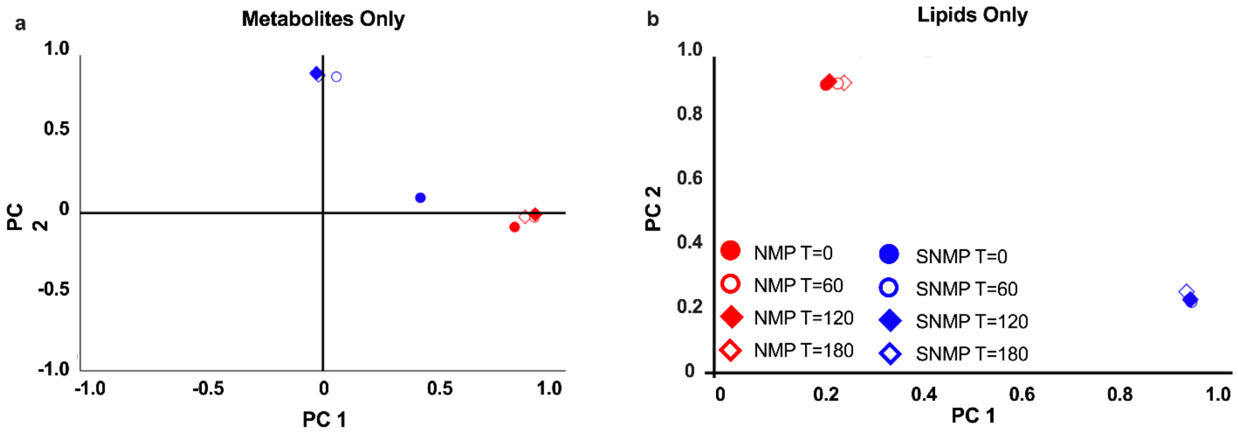

Principle component analysis of steatotic livers during NMP (blue) and SNMP (red) based on tissue biopsies taken at pre-perfusion (filled circle), 60 min (open circle), 120 min (filled diamond), and 180 min (open diamond). a) 2-dimensional representation of untargeted metabolomic profiles demonstrate similar pre-perfusion profiles of NMP and SNMP steatotic livers but group-wise clustering after initiation of perfusion. b) 2-dimensial representation of untargeted lipidomic profiles demonstrates group-wise clustering of steatotic livers during SNMP and NMP at all time points. NMP, normothermic machine perfusion; SNMP, subnormothermic machine perfusion; PC, principle component.

Figure S2 Heatmap of Metabolites Involved in Tricyclic Acid Cycle and Glycogen Metabolism

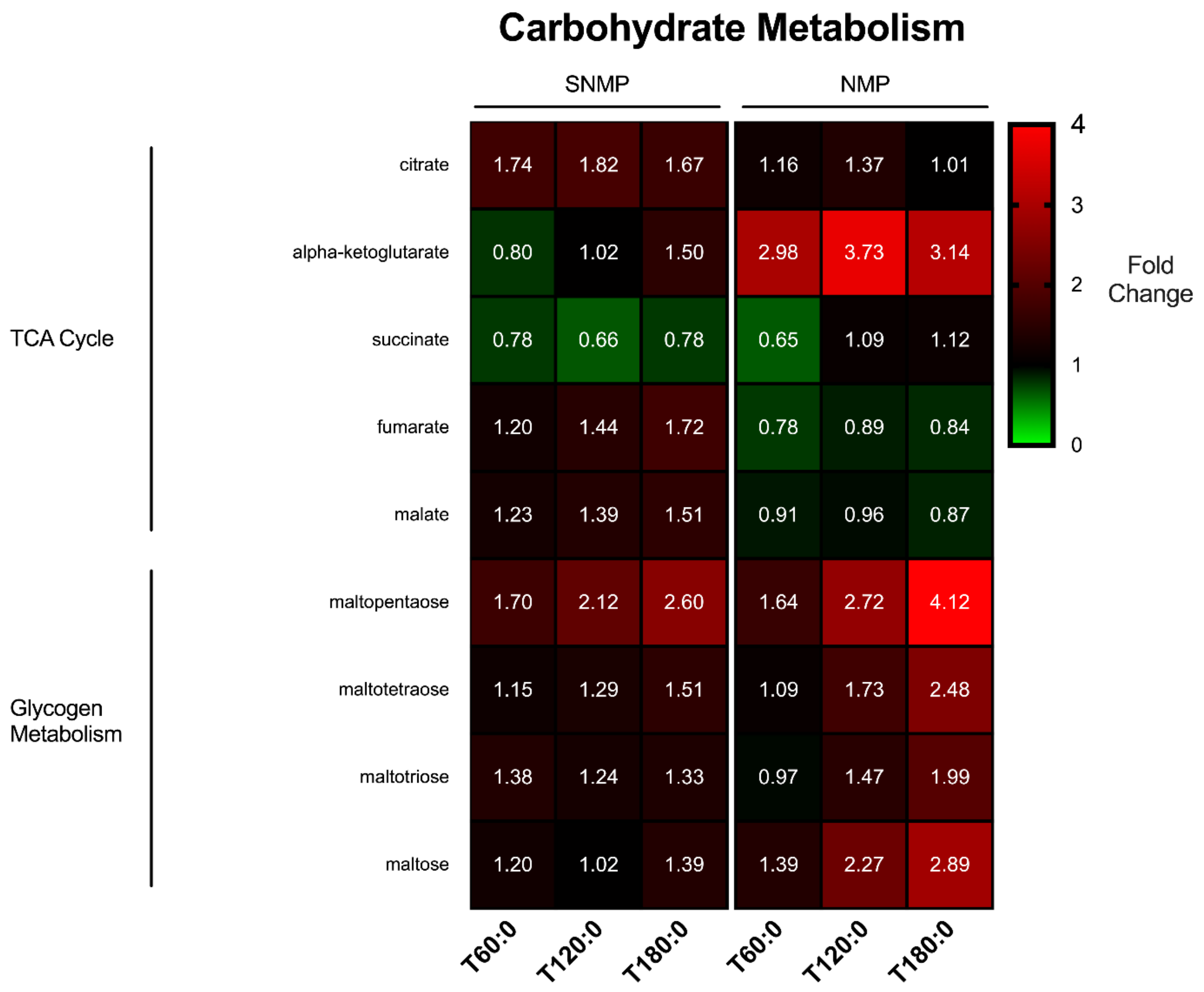

Metabolites in the tricyclic acid cycle (TCA) of SNMP livers demonstrate an overall accumulation compare to a relative decrease in NMP livers. Lower metabolic activity at subnormothermic temperatures results in less TCA activity versus active energy metabolism in during NMP. Glycogen components accumulate in both groups, but reach higher concentration ratios during NMP. NMP, normothermic machine perfusion; SNMP, subnormothermic machine perfusion; x-axis represents fold change at 60, 120, and 180 minutes compared to pre-perfusion concentrations.

Figure S3 Pathway Enrichment Analysis Comparing Pre-Perfusion Metabolomic Profiles of Steatotic Livers in SNMP versus NMP Group

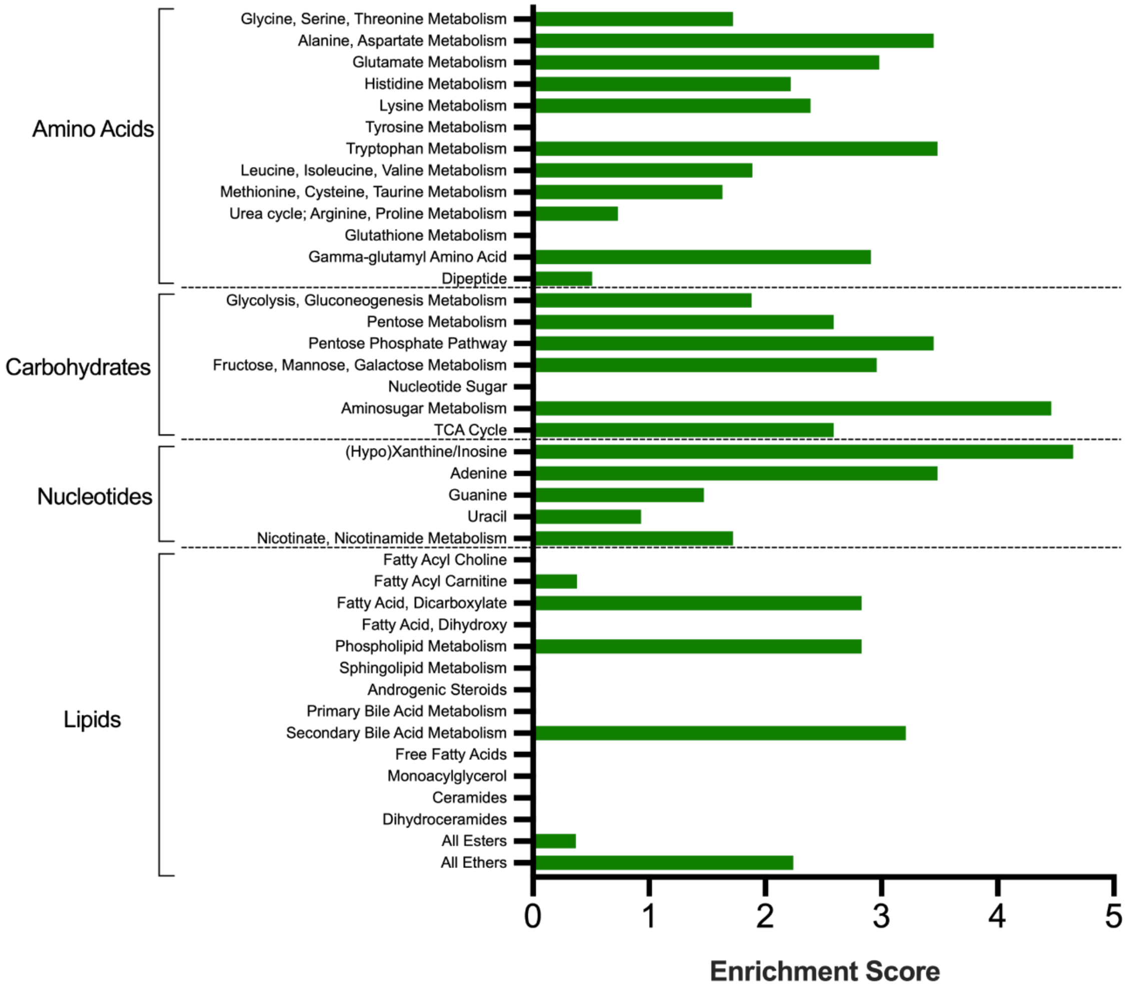

Pathway enrichment analysis comparing metabolomic profiles in pre-perfusion tissue samples from steatotic livers subjected to SNMP versus NMP demonstrates that the lipid profiles are largely similar between the two groups. Notably, metabolites in the glutathione metabolism pathway demonstrate no enrichment, indicating similar metabolite concentrations prior to initiation of perfusion in both groups. Pathways involved in amino acid and carbohydrate metabolism demonstrate enrichment between the groups prior to initiation of perfusion, likely as a result of procurement and donor differences.

3. Supplementary Methods

3S.1 Untargeted Metabolomic and Lipidomic Analysis (Performed by Metabolon, Inc.)

3S.1.1Sample Preparation

Samples were prepared using the automated MicroLab STAR® system from Hamilton Company. Several recovery standards were added prior to the first step in the extraction process for QC purposes. To remove protein, dissociate small molecules bound to protein or trapped in the precipitated protein matrix, and to recover chemically diverse metabolites, proteins were precipitated with methanol under vigorous shaking for 2 min (Glen Mills GenoGrinder 2000) followed by centrifugation. The resulting extract was divided into five fractions: two for analysis by two separate reverse phase (RP)/UPLC-MS/MS methods with positive ion mode electrospray ionization (ESI), one for analysis by RP/UPLC-MS/MS with negative ion mode ESI, one for analysis by HILIC/UPLC-MS/MS with negative ion mode ESI, and one sample was reserved for backup. Samples were placed briefly on a TurboVap® (Zymark) to remove the organic solvent. The sample extracts were stored overnight under nitrogen before preparation for analysis.

3S.1.2Quality Control (QC)

Several types of controls were analyzed in concert with the experimental samples: a pooled matrix sample generated by taking a small volume of each experimental sample (or alternatively, use of a pool of well-characterized human plasma) served as a technical replicate throughout the data set; extracted water samples served as process blanks; and a cocktail of QC standards that were carefully chosen not to interfere with the measurement of endogenous compounds were spiked into every analyzed sample, allowed instrument performance monitoring and aided chromatographic alignment. Instrument variability was determined by calculating the median relative standard deviation (RSD) for the standards that were added to each sample prior to injection into the mass spectrometers. Overall process variability was determined by calculating the median RSD for all endogenous metabolites (i.e., non-instrument standards) present in 100% of the pooled matrix samples. Experimental samples were randomized across the platform run with QC samples spaced evenly among the injections.

3S.1.3 Ultrahigh Performance Liquid Chromatography-Tandem Mass Spectroscopy (UPLC-MS/MS)

All methods utilized a Waters ACQUITY ultra-performance liquid chromatography (UPLC) and a Thermo Scientific Q-Exactive high resolution/accurate mass spectrometer interfaced with a heated electrospray ionization (HESI-II) source and Orbitrap mass analyzer operated at 35,000 mass resolution. The sample extract was dried then reconstituted in solvents compatible to each of the four methods. Each reconstitution solvent contained a series of standards at fixed concentrations to ensure injection and chromatographic consistency. One aliquot was analyzed

using acidic positive ion conditions, chromatographically optimized for more hydrophilic compounds. In this method, the extract was gradient-eluted from a C18 column (Waters UPLC BEH C18-2.1x100 mm, 1.7 µm) using water and methanol, containing 0.05% perfluoropentanoic acid (PFPA) and 0.1% formic acid (FA). Another aliquot was also analyzed using acidic positive ion conditions, however it was chromatographically optimized for more hydrophobic compounds. In this method, the extract was gradient eluted from the same aforementioned C18 column using methanol, acetonitrile, water, 0.05% PFPA and 0.01% FA and was operated at an overall higher organic content. Another aliquot was analyzed using basic negative ion optimized conditions using a separate dedicated C18 column. The basic extracts were gradient eluted from the column using methanol and water, however with 6.5mM ammonium bicarbonate at pH 8. The fourth aliquot was analyzed via negative ionization following elution from a HILIC column (Waters UPLC BEH Amide 2.1x150 mm, 1.7 µm) using a gradient consisting of water and acetonitrile with 10mM ammonium formate, pH 10.8. The MS analysis alternated between MS and data-dependent MS^n scans using dynamic exclusion. The scan range varied slighted between methods but covered 70-1000 m/z.

3S.1.4 TrueMass® Lipomic Panel

Lipids were extracted in the presence of authentic internal standards by the method of Folch et al. (*J Biol Chem* **226**:497-509) using chloroform:methanol (2:1 v/v). For the separation of neutral lipid classes [FFA, TAG, DAG, CE], a solvent system consisting of petroleum ether/diethyl ether/acetic acid (80:20:1) was employed. Individual phospholipid classes within each extract [PC, PE] were separated using the Agilent Technologies 1100 Series LC. Each lipid class was transesterified in 1% sulfuric acid in methanol in a sealed vial under a nitrogen atmosphere at 100°C for 45 minutes. The resulting fatty acid methyl esters were extracted from the mixture with hexane containing 0.05% butylated hydroxytoluene and prepared for gas chromatography (GC) by sealing the hexane extracts under nitrogen. Fatty acid methyl esters were separated and quantified by capillary GC (Agilent Technologies 6890 Series GC) equipped with a 30 m DB 88 capillary column (Agilent Technologies) and a flame ionization detector.

3S.1.5 Bioinformatics

The informatics system consisted of four major components, the Laboratory Information Management System (LIMS), the data extraction and peak-identification software, data processing tools for QC and compound identification, and a collection of information interpretation and visualization tools for use by data analysts.

3S.1.6 Data Extraction and Compound Identification

Raw data was extracted, peak-identified and QC processed using Metabolon's hardware and software. Compounds were identified by comparison to library entries of purified standards or

recurrent unknown entities. Metabolon maintains a library based on authenticated standards that contain the retention time/index (RI), mass to charge ratio (*m/z*), and chromatographic data (including MS/MS spectral data) on all molecules present in the library. Furthermore, biochemical identifications are based on three criteria: retention index within a narrow RI window of the proposed identification, accurate mass match to the library +/- 10 ppm, and the MS/MS forward and reverse scores between the experimental data and authentic standards. The MS/MS scores are based on a comparison of the ions present in the experimental spectrum to the ions present in the library spectrum. While there may be similarities between these molecules based on one of these factors, the use of all three data points can be utilized to distinguish and differentiate biochemicals.

3S.1.7 Curation

A variety of curation procedures were carried out to ensure that a high quality data set was made available for statistical analysis and data interpretation. The QC and curation processes were designed to ensure accurate and consistent identification of true chemical entities, and to remove those representing system artifacts, mis-assignments, and background noise. Metabolon data analysts use proprietary visualization and interpretation software to confirm the consistency of peak identification among the various samples. Library matches for each compound were checked for each sample and corrected if necessary.

3S.1.8 Metabolite Quantification and Data Normalization

Peaks were quantified using area-under-the-curve. For studies spanning multiple days, a data normalization step was performed to correct variation resulting from instrument inter-day tuning differences. Essentially, each compound was corrected in run-day blocks by registering the medians to equal one (1.00) and normalizing each data point proportionately (termed the "block correction"). For studies that did not require more than one day of analysis, no normalization is necessary, other than for purposes of data visualization. In certain instances, biochemical data may have been normalized to an additional factor (e.g., cell counts, total protein as determined by Bradford assay, osmolality, etc.) to account for differences in metabolite levels due to differences in the amount of material present in each sample.
The present dataset comprises a total of 1600 compounds of known identity. Following log transformation and imputation of missing values, if any, with the minimum observed value for each compound, ANOVA contrasts were used to identify biochemicals that differed significantly between experimental groups. An estimate of the false discovery rate (*q*-value) is calculated to take into account the multiple comparisons that normally occur in metabolomic-based studies. Standard statistical analyses are performed in ArrayStudio (OmicSoft Corp., Cary, NC, USA) on log transformed data. For those analyses not standard in ArrayStudio, the programs R (http://cran.r-project.org/) or JMP (Cary, NC, USA) are used.

3S.2 Principal Component Analysis (Performed by Manuscript Authors)

The metabolomic and lipidomic data for hourly tissue biopsies were projected onto principal components to enable visualization of the overall metabolic shift during perfusion for the 2 groups. The T = 0, 60, 120, and 180 minute time points were treated as independent, resulting in 8 observations. Principal component analysis (PCA) was constructed with rows as metabolites or lipids, columns as observation time points, and was computed using SPSS (IBM, Armonk, New York). Normalized data of each time point was used to perform PCA, which prevented the data with significantly higher average peak heights from dominating relative contribution towards the principal components (Supplemental Fig. 1a-b).
